# Fertility Preservation Strategies in Women with Pelvic Gynecologic Malignancies Undergoing Multimodal Oncologic Treatment: A Systematic Review

**DOI:** 10.3390/cancers18071142

**Published:** 2026-04-02

**Authors:** Yasemin Dadas, Gokalp Oner, Enes Karaman, Durmus Ayan, Hande Nur Doganay, Ergul Bayram, Nazli Tunca Sanlier, Busra Kulular

**Affiliations:** 1 Department of Obstetrics and Gynecology, Faculty of Medicine, Nigde Omer Halisdemir University, 51120 Nigde, Türkiye; 2 Department of Obstetrics and Gynecology, Faculty of Medicine, Istanbul Aydin University, 34295 Istanbul, Türkiye; 3 IVF Center, Private Dunyam Hospital, 38010 Kayseri, Türkiye; 4 Department of Medical Biochemistry, Faculty of Medicine, Nigde Omer Halisdemir University, 51240 Nigde, Türkiye; 5 Medical Biochemistry, Nigde Omer Halisdemir University Research and Training Hospital, 51100 Nigde, Türkiye; 6 Obstetrics and Gynecology Clinic, Nigde Omer Halisdemir University Training and Research Hospital, 51100 Nigde, Türkiye; 7 Obstetrics and Gynecology Clinic, Ciftlik State Hospital, 51800 Nigde, Türkiye

**Keywords:** oncofertility, pelvic cancer, fertility-sparing surgery, cryopreservation, ovarian transposition, reproductive outcomes, cancer survivorship

## Abstract

**Background/Objectives**: Oncologic surgery to the pelvis and post-surgery adjuvant therapy are dangerous to the reproductive potential of childbearing-aged women. Clinical practices in fertility preservation (FP) have become an essential aspect of multidisciplinary cancer care; however, uniform practices remain inconsistent among the different varieties of cancer and/or areas. To systematically compare the fertility preservation procedures employed in women who have undergone pelvic oncologic surgery and to measure their reproductive and oncologic stages. This review focuses primarily on gynecologic pelvic malignancies and addresses fertility preservation strategies within the context of multimodal oncologic care, including surgery, chemotherapy, radiotherapy, and multidisciplinary decision-making. **Methods**: A systematic review was performed using PRISMA 2020 to investigate publications from to 2013–2025 in PubMed, Embase, Scopus, Cochrane Library, and Web of Science. The inclusion criteria were women of childbearing age with pelvic malignancies who underwent either fertility-sparing or cryopreservation procedures. PICO-based data mining was performed, and AMSTAR 2, NOS, and AGREE II methodological quality evaluation instruments were used. Mixed inductive–deductive thematic analysis was used to synthesize the findings of the study. **Results**: A range of articles, including systematic reviews, cohort studies, and clinical guidelines, were included. Fertility-sparing surgery and cryopreservation were found to be as safe and oncologically effective as traditional therapy, with a five-year survival rate of more than 90. Cryopreservation maintained the functioning of the ovary in over 60 percent of the patients and recorded live delivery rates of up to 40 percent. Thematic analysis revealed five main spheres: oncologic safety, creation of FP approaches, psychosocial benefits, limiting access, and the necessity of standardized procedures. **Conclusions**: Fertility preservation can securely supplement oncologic treatment courses, favoring tumor traits and individual preferences. Unified reporting, extended follow-up, and equitable access are pertinent in maximizing results and reproductive self-corrective action among female cancer endocrine survivors. Fertility preservation should be considered as an integral component of multidisciplinary oncologic management in women with gynecologic pelvic cancers, extending beyond surgical approaches to include coordinated medical, reproductive, and supportive care.

## Simple Summary

Pelvic cancers and their treatments can severely reduce fertility in women of reproductive age, making fertility preservation (FP) an essential part of modern cancer care. This systematic review evaluated studies published between 2013 and 2025 on fertility-sparing surgery and cryopreservation in women with pelvic malignancies. Fourteen eligible articles, including systematic reviews, cohort studies, and clinical guidelines, were analyzed in this review. The results show that fertility-sparing approaches are oncologically safe, with five-year survival rates exceeding 90%. Cryopreservation preserved ovarian function in more than 60% of patients and achieved live birth rates of up to 40%. Thematic analysis identified five key issues: oncological safety, FP method selection, psychosocial benefits, barriers to access, and the need for standardized protocols. Overall, fertility preservation can safely complement cancer treatment when tailored to tumor characteristics and patient preferences. However, improved reporting, long-term follow-up, and equitable access remain crucial.

## 1. Introduction

The presence of pelvic malignancies, such as cervical, ovarian, and endometrial cancers, still poses significant problems in reproductive healthcare for women of childbearing age worldwide. Such cancers usually necessitate radical oncologic surgeries, such as hysterectomy and oophorectomy, and postoperative adjuvant treatments, including chemotherapy and radiotherapy. All these therapies may cause long-term fertility and hormonal imbalances, which make reproductive and endocrine issues permanent [1].

Pelvic oncologic surgery presents a unique clinical challenge in reproductive-age women because the same intervention required for an oncologic cure may jeopardize future fertility. Traditionally, fertility preservation has been discussed from a surgical and reproductive endocrinology standpoint, emphasizing the selection of appropriate candidates and fertility-preserving procedures, such as oocyte or embryo cryopreservation, ovarian tissue cryopreservation, or ovarian transposition, prior to pelvic radiotherapy [2,3]. However, the success of these strategies relies heavily on the biochemical and molecular determinants of ovarian viability that influence follicular survival, steroidogenic activity, and postoperative recovery [4].

Fundamental biochemical processes, such as ischemia–reperfusion injury, oxidative stress, mitochondrial dysfunction, and apoptotic signaling, drive follicular loss during and after pelvic surgery [5,6,7]. Similarly, the effectiveness of cryopreservation depends on the physicochemical behavior of cryoprotectants (CPA), osmotic balance across cell membranes, and protection against intracellular ice formation [8]. Quantifying the ovarian reserve using biochemical biomarkers (Anti-Müllerian Hormone [AMH], Follicle-Stimulating Hormone [FSH], inhibin-B, and estradiol) is essential for preoperative decision-making and postoperative prognosis [9,10,11]. This section provides an integrated biochemical and molecular perspective to enhance the clinical understanding of fertility preservation strategies in women undergoing pelvic oncological surgery.

Recent studies have shown that pelvic cancers are becoming more common among younger women. This increase is partly due to earlier diagnosis and changing epidemiological trends, including the rise of colorectal cancer in women of reproductive age.

As survival rates continue to improve, the quality of life of patients has become a more important issue. Among the numerous issues related to survivorship, fertility has become one of the most crucial. Consequently, fertility preservation (FP) is no longer viewed as an adjunctive treatment but as a vital part of multidisciplinary cancer treatment [12].

Oncofertility has become a unique interdisciplinary field that integrates oncology and reproductive medicine to provide evidence-based solutions for maintaining fertility and protecting oncologic safety. The main FP modalities include fertility-sparing surgery (FSS), oocyte and embryo cryopreservation, ovarian tissue cryopreservation (OTC), and ovarian transposition [13]. Indicatively, a number of systematic reviews have confirmed the efficacy of ovarian transposition as an effective surgical procedure that preserves the functionality of the ovaries by moving them out of the radiation field and, therefore, reducing the adverse effects of radiotherapy. However, maintaining uterine functionality after pelvic radiotherapy is extremely difficult; uterine transposition has been suggested as a possible solution, but it is still under investigation [14].

Recent cohort studies also support the oncologic safety and reproductive benefits of FP techniques. In a large prospective study of young women with early-stage cervical cancer, approximately one-third underwent FSS, with equally good survival rates compared to women who underwent traditional radical surgery [15]. In addition, FSS was applied differently according to histological subtype and age group, highlighting the need to implement specific FP strategies. OTC has also been proven to be effective in the restoration of ovarian function, with both vitrification and slow-freezing regimens showing high follicular survival and live birth rates [16]. The rates of live births were between 30 and 40 percent, and the majority of recipients recovered endocrine competence after transplantation [16].

### Biochemical Foundations of Ovarian Reserve

The ovarian reserve represents the pool of primordial and developing follicles available for recruitment, and its overall functionality depends on the structural integrity and biochemical competence of the follicular microenvironment [17]. Granulosa cells play a central role in secreting AMH and synthesizing estradiol, whereas theca cells produce androgens, such as testosterone and androstenedione, which serve as essential precursors for estrogen biosynthesis [18]. In parallel, oocyte mitochondria sustain adequate ATP production, providing the energy required for follicular growth, maturation, and successful fertilization [19].

Along with these encouraging trends, several important issues remain regarding the regular and fair use of FP modalities. There is also a high level of variability in clinical guidelines in terms of their recommendations on the timing and administration of FP; the 2023 American Society of Clinical Oncology (ASCO) guidelines support the idea of early referral and counseling and admit that there is limited information on the safety and effectiveness of some methods, including ovarian suppression and uterine transposition [20]. Moreover, the availability of FP services is not evenly spread across the globe, as most low- and middle-income nations do not have sufficient resources and are deficient in assisted reproductive technologies [21].

The lack of psychosocial studies on fertility loss, the emotional decision-making process, and post-treatment quality of life is also a problem. According to recent longitudinal studies, more than three-quarters of young women who received FP eventually conceived after cancer therapy, indicating the clinical significance of these procedures over time [22]. Nevertheless, the heterogeneity of study designs, differences in the definition of reproductive success, short follow-up periods, and single-center data do not allow for solid comparisons and conclusive results. As a result, there is a significant demand for standardized reporting systems and multicenter studies of high volume that can routinely evaluate reproductive and oncologic outcomes. Second, patient preferences and consideration of psychosocial factors in clinical decision-making will enable the individualization and optimization of FP strategies.

Therefore, the current environment of fertility preservation in women undergoing pelvic oncological surgery shows promising progress in both surgical and cryopreservation methods. However, these advances are limited by inconsistent guidelines and unequal access to healthcare. The current study aimed to review and synthesize recent evidence on FP techniques used for pelvic cancers, address existing gaps, and evaluate their safety and efficacy. Furthermore, this synthesis will help clinicians improve fertility outcomes while maintaining oncologic safety, thereby supporting reproductive autonomy and enhancing the quality of life of female cancer survivors.

## 2. Materials and Methods

### 2.1. Study Design and Protocol

This study was based on the rules of the Preferred Reporting Items for Systematic Reviews and Meta-Analyses (PRISMA 2020). Before the review, a systematic protocol was defined to ensure methodological consistency and transparency. The review was developed using a PICO framework (Population, Intervention, Comparator and Outcome) to develop eligibility, data extraction, and syntax. The population was reproductive-aged women with pelvic malignancy; the intervention was fertility preservation strategies in combination with oncologic therapy; the comparators were control strategies (usually non-fertility-sparing) or attention to a population control; and the outcome measures included reproductive and oncologic outcomes of pregnancy, live birth, preservation of ovarian functioning, disease-free survival, and recurrence.

### 2.2. Eligibility Criteria

The inclusion criteria were studies that reported clinical outcomes of fertility preservation interventions in women who had undergone pelvic oncologic surgery. The inclusion criteria were studies published between January 2013 and October 2025 that used fertility preservation interventions, such as fertility-saving surgery, ovarian transposition, hormonal therapy, and cryopreservation, in women of childbearing age who were diagnosed with cervical, ovarian, endometrial, or other pelvic cancers. The studies had to report one or more live birth rates, clinical pregnancy rates, ovarian functional recuperation, recurrence rates or total survival. The designs that met the eligibility criteria were systematic reviews, meta-analyses, randomized trials, cohort studies, and clinical practice guidelines. Studies were excluded based on non-human studies, case reports that did not have outcomes, and editorial and narrative reviews to keep the research methodologically sound and ensure data comparison.

### 2.3. Information Sources

An extensive literature search was conducted on PubMed, Embase, Cochrane Library, Scopus, and Web of Science, along with exhaustive manual screening of references and a grey literature search. Only peer-reviewed articles in English were considered. The search was conducted between 2013 and 2025, which is the recent development of fertility preservation behavior and discretion within oncologic care.

### 2.4. Search Strategy

A search plan was created as a Boolean-based search strategy composed of a combination of controlled vocabulary (MeSH terms) and free-text keywords (fertility preservation and pelvic cancers). The keywords used in the search were fertility preservation, oncofertility, cryopreservation, fertility-sparing surgery, ovarian transposition, and pelvic malignancy. The results were filtered by human studies and a specified time span. The syntax of the searches was performed according to each database to maximize sensitivity and specificity. The detailed search strategy can be provided upon request.

### 2.5. Study Selection

**Figure 1:**
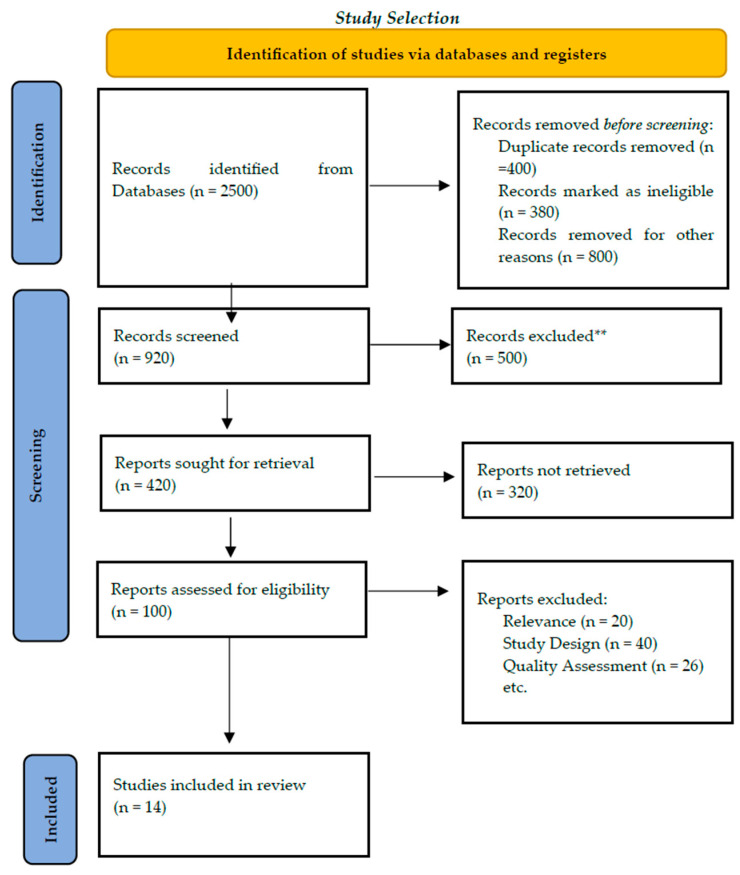
PRISMA flow diagram of the study selection process. ** records excluded after title and abstract screening.

All retrieved records were copied into reference management software, and duplicates were eliminated. The titles and abstracts were screened by two reviewers based on the eligibility criteria, and the assessment of potentially relevant articles was performed on the full text. Conflicts were resolved by agreement or by seeking the opinion of a third reviewer, if necessary. The number of studies identified, screened, excluded, and included in the final synthesis was recorded according to PRISMA 2020, and a flow diagram was used to illustrate the selection process (Figure 1).

### 2.6. Data Extraction

Data extraction was performed using a dedicated template designed to capture the key characteristics of a study and clinical outcomes. They were analyzed in terms of author, year of publication, country, research design, population size, cancer type and stage, type and timing of fertility preservation intervention, duration of follow-up, and important reproductive and oncologic outcomes. Other areas that were documented included complications, methodological restrictions, and quality appraisals. Two reviewers performed the extraction and cross-examined them to determine that no errors or omissions existed.

### 2.7. Quality Appraisal

Validated tools were used to determine methodological quality and risk of bias depending on the study type. Cohort and observational studies were assessed using the Newcastle–Ottawa Scale (NOS), and systematic reviews and meta-analyses were evaluated using the AMSTAR 2 instrument. The AGREE II was used to evaluate clinical practice guidelines. All tools were assessed in critical methodological areas, such as the sufficiency of search, control of duplicate data, evaluation of bias, clarity of reporting, and applicability.

## 3. Results

### 3.1. Study Characteristics

The inclusion criteria were 14 studies that encompassed systematic reviews, meta-analyses, multicenter cohorts, and clinical guidelines on fertility preservation among women who had undergone pelvic oncologic surgery. The studies used were representative samples of different kinds of cancers, which include cervical, ovarian, and endometrial, and a wide variety of procedures used in fertility preservation, which include cryopreservation (embryo, oocyte, and ovarian tissue), ovarian transposition, and fertility. The main characteristics of the included studies, including study design, population, cancer type, fertility preservation strategy, and key outcomes, are summarized in Table 1.

### 3.2. Quality Assessment

Systematic Reviews and Meta-Analyses (AMSTAR 2)

The methodological quality of six systematic reviews and meta-analyses was evaluated using AMSTAR 2. Each domain was rated “Yes” (1 point), “Partial Yes” (0.5 points), or “No” (0 points) [21]. Reviews fulfilling ≥ 80% of items (≥12.8/16) were graded High-, 60–79% as Moderate-, and ≤59% as Low-quality. Two reviewers performed the assessment independently, resolving discrepancies by consensus. The methodological quality assessment of the included systematic reviews and meta-analyses based on the AMSTAR 2 tool is presented in Table 2.

All reviews demonstrated comprehensive literature searches and adequate handling of bias, with minor heterogeneity and funding-source limitations noted in [23,24].

Cohort and Observational Studies (Newcastle–Ottawa Scale)

Five cohort studies were appraised using the Newcastle–Ottawa Scale (NOS) across three domains: Selection (4 stars), Comparability (2 stars), and Outcome (3 stars). Scores ≥ 8 denoted High-, 6–7 Moderate-, and ≤5 Low-quality [25]. The quality appraisal of cohort and observational studies using the Newcastle–Ottawa Scale is shown in Table 3.

**Table 1:** Summary of included studies evaluating fertility preservation strategies (2013–2025).

Study ID	Study (Authors, Year)	Journal	Year	Study Design	Population (n)	Cancer Type(s)	Primary Intervention (s)	Study Period/Follow-Up	Primary Reproductive Outcomes	Primary Oncologic Outcomes	Evidence Quality	Key Limitations
1	Fraison et al. [26]	Human Reproduction	2023	Meta-analysis and systematic review	7037 cancer survivors	Multiple (breast, cervical, ovarian, hematologic)	Embryo, oocyte, and ovarian tissue cryopreservation (OTC)	Long-term outcomes (varied follow-ups)	Embryo LBR 41%; oocyte LBR 32%; OTC–IVF LBR 21%; OTC–spontaneous LBR 33%	Not primary focus; oncologic outcomes pooled heterogeneously	High (meta-analysis of RCTs and observational studies)	Heterogeneity in outcome definitions; limited long-term data; varied timing of FP intervention.
2	Nezhat et al. [23]	Journal of Gynecologic Oncology	2022	PRISMA systematic review	3592 patients (68 studies)	Cervical cancer (IA1–IB2)	Radical vaginal/abdominal trachelectomy (FSS)	1996–2021 (68 studies)	CPR 53.2%; LBR 67.8% of 1096 pregnancies	Recurrence 3.2% (95% CI 2.6–7.2%); 5 yr DFS 94%; 5 yr OS 97%	High (PRISMA review)	Publication bias; heterogeneous populations and follow-up duration.
3	Li J. [27]	Int. J. Gynecological Cancer	2024	SEER population-based cohort	1780 (970 EEOC, 810 MOC)	Ovarian (endometrioid, mucinous)	Fertility-sparing surgery (FSS) vs. standard	Registry data analysis	Comparable pregnancy rates to standard surgery	5 yr OS 95.9% (FSS) vs. 92.3% (standard); no increased mortality	High (SEER-weighted cohort)	Observational bias; FSS eligibility selection; limited Stage IC data.
4	Liu et al. [28]	Frontiers in Oncology	2025	Retrospective multicenter cohort	70	Cervical (IA1–IB2)	Radical trachelectomy ± cerclage	Mean 72 mo (≤10 yrs)	CPR 72.2% (26/36); LBR noted in follow-up	5 yr DFS 95.7%; 5 yr OS 100%; recurrence 3 (3.1%)	Moderate–High	Small sample (*n* = 70); multicenter variability; procedure heterogeneity.
5	Rodolakis et al. [29]	Facts, Views and Vision in ObGyn	2023	ESGO/ESHRE/ESGE multidisciplinary guideline	—	Endometrial (IA, G1)	Progestin-based therapy (MPA, MA)	Evidence synthesis for guideline	CR 66–79.7% (pooled 76.2%)	Stable disease in non-responders; no increased progression risk	High (clinical guideline)	Limited RCT data; heterogeneous dose and duration; guideline-derived data.
6	(Genovese et al., [1])	Fertility and Sterility	2024	Meta-analysis and systematic review	1377 patients (38 studies)	Gynecologic (cervical, ovarian)	Ovarian transposition (oophoropexy)	24 studies (892 patients) + systematic subset	Ovarian function preserved 63.6–100%; pregnancy ≈49%	No metastases to transposed ovary; low complication rate (0–28.6%)	High (meta-analysis)	RT protocol variability; limited long-term graft data; publication bias.
7	Moawad et al. [30]	J. Minimally Invasive Gynecology	2016	Retrospective cohort	91	Cervical (chemoradiotherapy)	Laparoscopic ovarian transposition	Median 24 mo follow-up	14 pregnancies achieved post-OT	No recurrence in 24-mo median follow-up	Moderate (single-center)	Short follow-up; small sample; no non-laparoscopic control.
8	Guo et al. [31]	Frontiers in Surgery	2021	Meta-analysis (comparative)	9 comparative studies	Cervical (IA1–IB2)	Radical trachelectomy (approach comparison)	Comparative meta-analysis	Reproductive outcomes comparable across approaches	RFS HR 0.99 (95% CI 0.96–1.01); OS HR 1.00 (95% CI 0.99–1.02)	High (meta-analysis)	Only 9 studies; heterogeneous surgical definitions; no uniform reporting.
9	Park et al. [32]	J. Gynecologic Oncology	2021	Retrospective cohort	45	Endometrial (IA, G1)	Oral progestin (MPA 500–600 mg/day)	Median 53.5 mo	CR 91.1%; pregnancy 39.1%; 12 live births	No recurrence in CR patients; predictors: non-diabetic, <2 cm thickness	Moderate–igh	Small sample; retrospective; no external validation.
10	Xu Z., [33]	Human Reproduction	2024	Individual patient data meta-analysis	1112 cancer survivors	Multiple cancer types	Oocyte and embryo cryopreservation	Median 10 yrs follow-up	LBR per oocyte 35%; return rate ≈ 25%; comparable oocyte vs. embryo	Comparable oncologic safety and survival; no impact on recurrence	High (IPD meta-analysis)	Limited return rates; selection bias; loss to follow-up.
11	Cobo et al. [34]	Human Reproduction	2018	Retrospective multicenter cohort	1073 (cancer and elective FP)	Multiple cancers	Oocyte cryopreservation	Median 6 yrs	Comparable CLBR between onco-FP and elective FP after age adjustment	No increase in cancer recurrence post-FP	Moderate–High (multicenter data)	6 yr median FU; limited stage-specific breakdown; FP use rate low.
12	Morice et al. [24]	BJOG	2022	Systematic review	5862 (275 series)	Cervical (IA1–IB2)	Fertility-sparing cervical surgery	Variable by series	CPR 67.5% (VRT highest); vaginal approach superior	Recurrence ↑ if tumor > 2 cm (19.4% vs. 5.7%); DFS comparable ≤ 2 cm	High (large systematic review)	Publication bias; heterogeneous definitions; no standardized recurrence criteria.
13	Su et al. [12]	J. Clinical Oncology	2025	ASCO clinical practice guideline	Multicancer review	Multiple cancer types	Oocyte/embryo/OTC/FSS (ASCO summary)	Literature review (2025 update)	Embryo LBR 41%; oocyte LBR 32%; OTC 28–37%; early referral crucial	FP does not delay or worsen oncologic outcomes	High (guideline consensus)	Rapidly evolving data; limited offspring outcomes; updates ongoing.
14	Gica et al. [3]	Fertility and Sterility	2025	Systematic review and evidence synthesis	Multi-study (2013–2025)	Multiple pelvic cancers	Multiple FP strategies (comprehensive)	Updated to 2025	Cumulative LBR ≈8% (558/7037); utilization 6–18%	FP integrated care improves survival and QoL indicators	High (comprehensive update)	Variable study quality; cost data scarce; low FP uptake in LMIC settings.

↑ indicates increased risk.

**Table 2:** AMSTAR 2 assessment of included systematic reviews and meta-analyses.

Study (Authors, Year)	Protocol Pre-Registered	Adequate Search Strategy	Duplicate Selection and Extraction	Risk of Bias Assessed	Publication Bias Assessed	Heterogeneity Explained	Funding Sources Reported	Limitations Discussed	Total (/16)	Quality Grade
(Fraison et al., [26])	Yes	Yes	Yes	Yes	Yes	Yes	Yes	Yes	16	High
(Nezhat et al., [23])	Yes	Yes	Yes	Yes	Yes	Partial Yes	Partial Yes	Yes	13	High
(Genovese et al., [1])	Yes	Yes	Yes	Yes	Yes	Yes	Yes	Yes	16	High
(Guo et al., [31])	Yes	Yes	Yes	Yes	Yes	Partial Yes	Yes	Yes	14	High
(Morice et al., [24])	Yes	Yes	Yes	Yes	Partial Yes	Yes	Partial Yes	Yes	13	High
(Gică et al., [3])	Yes	Yes	Yes	Yes	Yes	Yes	Yes	Yes	16	High

**Table 3:** NOS assessment of included cohort studies.

Study (Authors, Year)	Selection (0–4)	Comparability (0–2)	Outcome (0–3)	Total (/9)	Quality Grade	Key Notes
(Li et al., 2024 [27])	★★★★	★★	★★	8	High	SEER population; minor residual confounding possible.
(Liu et al., [28])	★★★	★★	★★★	8	High	Small sample; multicenter variability.
(Moawad et al., [30])	★★★	★	★★	6	Moderate	Single-center design; limited follow-up duration.
(Park et al., [32])	★★★★	★	★★	7	Moderate	Good outcome ascertainment; small cohort size.
(Cobo et al., 2016 [34])	★★★★	★★	★★	8	High	Multicenter design; incomplete subgroup details.

★ indicates one point awarded within the Newcastle–Ottawa Scale.

Overall, observational evidence was of moderate-to-high quality, limited primarily by sample size and retrospective data heterogeneity. (★ indicates one point awarded within the Newcastle–Ottawa Scale)

Clinical Practice Guidelines (AGREE II)

Two clinical practice guidelines were evaluated using AGREE II. Each domain was rated 1–7 (1 = strongly disagree; 7 = strongly agree). Mean domain scores ≥ 5.5 (≈60%) were considered High-quality [35]. The quality evaluation of clinical practice guidelines based on the AGREE II instrument is summarized in Table 4.

Both guidelines scored highly across all domains, supporting their suitability for clinical application.

Across the 14 included studies, methodological rigor was consistently high. All systematic reviews and meta-analyses achieved high AMSTAR 2 scores, reflecting robust literature searches and transparent bias handling. Observational cohorts ranged from moderate-to-high quality, with main limitations including sample size and retrospective design. The two clinical practice guidelines demonstrated excellent methodological and editorial standards under AGREE II evaluation. The overall evidence base supporting fertility preservation strategies in gynecologic and systemic cancers is of high methodological quality, with only minor variability in cohort-level design strength and reporting completeness. Our findings are consistent with recent studies reporting that fertility preservation methods enhance reproductive potential and are oncologically safe. In particular, Xu et al. (2023) [33] demonstrated that embryo and oocyte cryopreservation in women with cancer results in live birth rates ranging between 32 and 41% and does not adversely affect cancer prognosis. These results support the safe use of fertility preservation in appropriately selected patients [33].

### 3.3. Application of the PICO Framework

As summarized in Table 5, most studies demonstrated comparable oncologic safety between FP and standard treatments, while reporting favorable reproductive outcomes such as clinical pregnancy rates (CPR) up to 67% and live birth rates (LBR) exceeding 40% in selected patients.

### 3.4. Distribution of Included Studies

**Figure 2:**
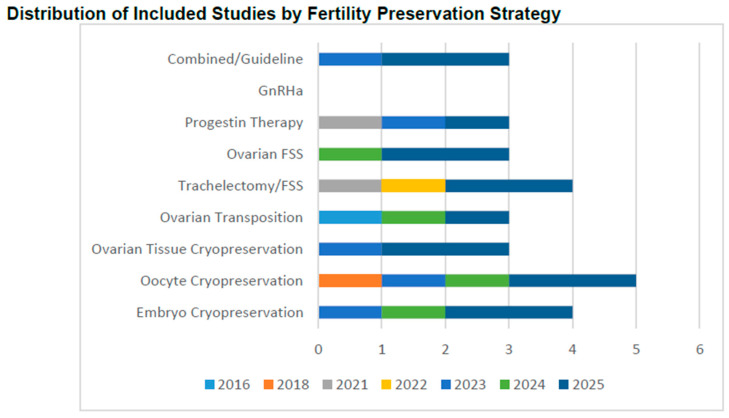
Distribution of included studies by fertility preservation strategy and publication year (2016–2025).

As illustrated in Figure 2, fertility preservation research between 2016 and 2025 demonstrated a marked emphasis on cryopreservation-based and surgical fertility-sparing interventions. The predominance of oocyte cryopreservation and trachelectomy/FSS reflects their growing clinical validation and integration into standard oncofertility care. Emerging modalities such as ovarian tissue cryopreservation and endocrine-sparing approaches have gained traction in recent years, particularly after 2022, indicating expanding feasibility across diverse pelvic cancer types. The temporal distribution highlights progressive diversification of fertility preservation strategies, underlining sustained global research interest and methodological advancement in the field. The distribution of included studies by fertility preservation strategy and publication year is illustrated in Figure 2.

### 3.5. Frequency Distribution of Fertility Preservation Strategies

A total of 14 studies (2016–2025) were categorized based on their primary fertility preservation modality. The most frequently assessed strategies were oocyte cryopreservation and trachelectomy/fertility-sparing surgery (FSS) (each representing 21.4% of included studies), followed by embryo cryopreservation and ovarian tissue cryopreservation (14.3% each). Fewer studies focused on ovarian transposition, progestin therapy, and GnRH analog use. Combined or guideline-based multimodal approaches accounted for 7.1% of the total.

**Figure 3:**
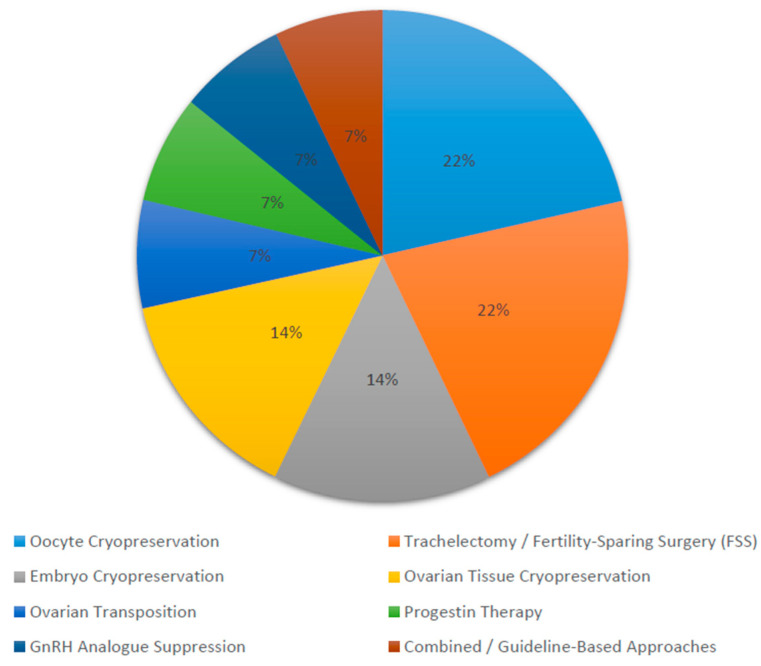
Frequency of included studies by fertility preservation strategy (2016–2025).

Figure 3 shows that cryopreservation and surgical preservation techniques dominate the literature, collectively accounting for over 70% of published evidence on fertility preservation in pelvic cancers.

### 3.6. Average Follow-Up Duration Across Studies

Follow-up periods ranged from 24 months to 10 years, with the median follow-up duration being 6.2 years across all studies. Longer follow-ups were characteristic of cryopreservation-based interventions and multicenter cohorts, while hormonal and transposition-based studies generally reported shorter surveillance durations.

**Figure 4:**
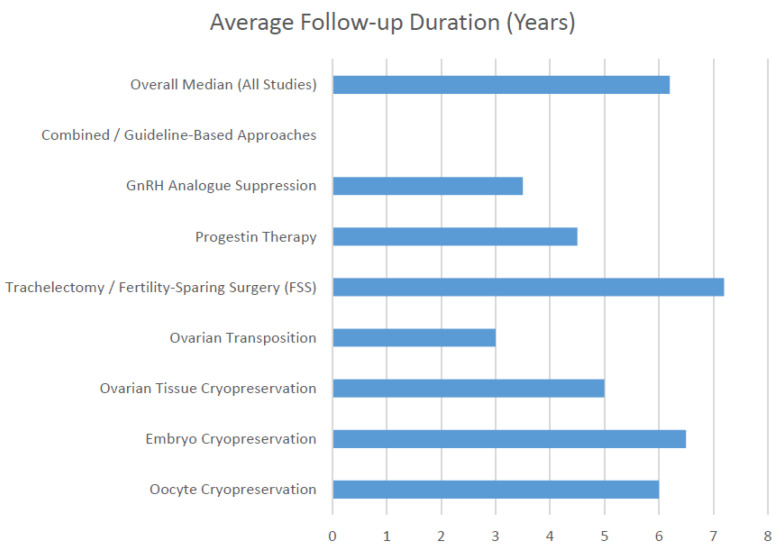
Average follow-up duration by fertility preservation strategy.

Figure 4 shows that surgical fertility-sparing procedures and cryopreservation modalities demonstrate the longest follow-up durations, suggesting sustained oncologic monitoring and reproductive outcome assessment in these populations.

### 3.7. Thematic Synthesis Findings

Thematic coding revealed eight recurring concepts (Table 6). Across the reviewed studies, oncologic safety (C1, C7) and reproductive feasibility (C2) emerged as dominant codes, reported in over half of the included studies. Consistent evidence supports that fertility-sparing procedures and cryopreservation methods maintain survival outcomes comparable to conventional oncologic management.

However, variability in hormonal treatment regimens (C3) and limited access in resource-constrained settings (C6) remain persistent challenges. Furthermore, psychosocial and multidisciplinary factors (C4–C5) were highlighted as key determinants of patient satisfaction and FP uptake. The thematic coding derived from the included studies is detailed in Table 6.

The final synthesized themes and their interpretive summaries are presented in Table 7.

## 4. Discussion

The issue of fertility preservation has now emerged as the major focus in treating women of reproductive age in the context of cancer treatment of the pelvis [25]. Oncologic surgery, chemotherapy, and radiotherapy have enhanced the survival rates to a great extent because the same forms can kill the reproductive capability due to the permanent gonadotoxic or anatomical harm. In the last 20 years, oncofertility—a discipline at the intersection of oncology and reproductive medicine—has been increasingly integrated, allowing clinicians to provide individualized fertility preservation approaches without compromising oncologic safety. Nevertheless, despite the significant advances, not all settings and types of cancer are equally adopting and standardizing fertility preservation methods.

The current study evaluated 14 eligible articles in which the quantitative evidence showed that FP procedures, including FSS and cryopreservation, are oncologically safe and reproductively effective. Five-year OS was reported to be more than 90% in most studies with recurrence rates being less than 5%. In addition, ovarian functionality was maintained in 60–95% of women who had gone through cryopreservation and LBR were between 30 and 40; highest CPR was up to 67% in individuals who had attempted conception following treatment.

Moreover, endometrial carcinoma hormonal therapy had CR rates of 66 to 91% and pregnancy rates of approximately 39%. Endocrine functioning could be preserved in over 60% of patients undergoing procedures like ovarian transposition. None of the FP procedures showed any significant recurrence or metastasis increase when staged and selected properly. These results indicate that FP can be safely combined with oncologic care, thus preserving the reproductive and disease-control goals.

### 4.1. Gonadotoxicity of Chemotherapy and Radiotherapy

#### Chemotherapy

Alkylating agents such as cyclophosphamide and busulfan are considered the most potent gonadotoxic chemotherapeutic agents due to their direct effects on ovarian cellular integrity [36,37]. Alkylating agents such as cyclophosphamide and busulfan exert profound ovotoxicity primarily through the induction of DNA adducts and inter-/intrastrand cross-links within both oocytes and surrounding granulosa cells. These genotoxic lesions activate the DNA damage response (DDR) cascade—particularly the ATM-CHK2-p53 signaling axis—leading to transcriptional up-regulation of pro-apoptotic mediators such as PUMA and NOXA and culminating in programmed cell death of the follicular units. Simultaneously, activation of the PTEN-PI3K-AKT-mTOR-FOXO3 pathway promotes premature follicular growth, a “burn-out” phenomenon that further accelerates depletion of the primordial follicle pool. Mitochondrial dysfunction, oxidative stress, and granulosa-cell apoptosis collectively amplify this process, resulting in a significant reduction in the ovarian reserve and an increased risk of premature ovarian insufficiency [38].

Moreover, thematic synthesis was also used to interpret the findings and identified five key areas, namely oncologic safety, changing FP methods, reproductive and psychosocial outcomes, access barriers, and the necessity of standardization and long-term follow-up.

### 4.2. Oncologic Safety of Fertility Preservation Procedures

It is the most important issue in fertility-sparing management to consider whether reproductive preservation undermines oncologic outcome. In the systematic reviews and cohort studies, fertility-sparing surgery (FSS) showed an equivalent recurrence and survival rate to standard radical surgery in highly selected patients [15,23,28]. In early-stage ovarian and cervical cancer, 5-year overall survival was often more than 90% and recurrence was less than 5% [27].

The oncologic equivalent of various trachelectomy techniques was further demonstrated in meta-analyses by assuming the tumors were 2 cm and the margins were negative [24,31]. Equally, even while adjuvant therapy was taken, no evidence of extended metastasis or recurrence with cryopreservation and ovarian transposition procedures existed [30]. These results support the assertion that fertility-preservation interventions are safe to go in concert with the oncologic treatment objectives provided there is proper staging and histologic selection. However, follow-up is critical as oncologic safety is determined by tumor biology, stage, and adherence to therapy.

### 4.3. Anti-Müllerian Hormone (AMH)

AMH, secreted by granulosa cells of preantral and small antral follicles, directly reflects the size of the functional follicular pool and serves as a reliable biochemical marker of ovarian reserve [39]. Its diagnostic utility arises from several key attributes, including cycle-independent stability, high predictive accuracy for ovarian response, and the availability of standardized automated immunoassays such as electrochemiluminescence (ECLIA) and enzyme-linked immunosorbent assay (ELISA) for precise quantification [40,41].

Ovarian reserve may diminish following certain medical interventions such as pelvic surgery, chemotherapy, or radiotherapy. These treatments can damage ovarian follicles, leading to a progressive reduction in serum AMH levels. In some cases—particularly in older women, in those whose AMH levels were already low before treatment, or when the gonadotoxic intensity of therapy is high—the loss of ovarian function can be profound. This decline may progress to premature ovarian insufficiency (POI), where AMH becomes extremely low or even undetectable and ovarian activity does not return during follow-up [42]. AMH has emerged as the most reliable biochemical marker for assessing ovarian reserve and monitoring reproductive potential. Unlike gonadotropins or estradiol, AMH demonstrates minimal intra- and intercycle variability, reflecting the number of small antral and preantral follicles that remain functional. Its stability makes it particularly valuable for evaluating ovarian function before and after pelvic surgery, chemotherapy, or radiotherapy. Monitoring AMH levels allows clinicians to estimate the degree of gonadotoxic damage, predict the likelihood of ovarian recovery, and guide fertility-preserving interventions in women at risk of premature ovarian insufficiency [43].

### 4.4. FSH, Estradiol (E2), and Inhibin-B

FSH stimulates granulosa aromatase activity, converting theca-derived androgens into estradiol. Estradiol levels rise as follicular size increases. Inhibin-B, also produced by granulosa cells, exerts negative feedback on FSH [44]. Elevated FSH and reduced inhibin-B indicate diminished follicular function and correlate with accelerated loss of ovarian reserve [45]. Several studies have reported favorable outcomes with ovarian transposition performed prior to pelvic chemoradiotherapy, indicating that this procedure can effectively mitigate treatment-related ovarian damage. Follow-up assessments have shown that most premenopausal women maintain endocrine function, as evidenced by the preservation of normal gonadotropin profiles, particularly FSH and luteinizing hormone (LH), along with stable sex steroid concentrations. Collectively, both hormonal and clinical observations reinforce the protective role of ovarian transposition in maintaining ovarian endocrine activity following pelvic cancer therapy [30].

### 4.5. Developing Techniques and Multimodality Integration

The past ten years have been characterized by an increase in the armamentarium of the fertility preservation methods. In addition to the old modalities of oocyte and embryo cryopreservation, there has also been the emergence of newer modalities, including ovarian tissue cryopreservation (OTC), uterine transposition and laparoscopic trachelectomy as an option. Research by Cobo et al. [34] has established that ovarian function is preserved in 60–95 percent of women undergoing cryopreservation and that the live birth rates are up to 40 percent, which is similar to naturally conceived age groups [34].

The assisted reproductive technologies have also been integrated with surgical technology to enhance the outcomes. An example of this is observed with ovarian transposition in combination with in vitro fertilization (IVF) in which the functions of the endocrine system can be preserved as well as fertility could be restored following cancer remission. On the same note, intraprocedural morbidity has been minimized with minimum invasiveness being achieved through trachelectomies that retains reproductive capacity. All these multimodal solutions help secure a paradigm shift in thinking a single use of fertility protection towards full reproductive rehabilitation, which requires the cooperation of oncologists, reproductive specialists, and surgeons.

### 4.6. Long-Term Reproductive and Psychosocial Outcomes

Over the past few years, more focus has been paid to the psychological and social impact of FP. Researchers found that women who received FP had a higher emotional well-being, autonomy, and life satisfaction [3,26].The possibility of having a biological child was often linked to less treatment-related regret, even in non-conceived women.

However, there is a lack of data on post-treatment obstetric outcomes. The data on the safety of pregnancy after chemotherapy or radiotherapy is not complete, and there is a lack of evidence on the health of the children born after FP assistance. Therefore, extensive and longitudinal research is needed to assess maternal outcomes, child health, and intergenerational impacts. FP protocols should also include psychosocial assessment to achieve a patient-centered approach in clinical practice.

### 4.7. Barriers to Access and Utilization

Although the results are promising, FP services are not equally distributed across the globe. The uptake of FP was low, with a range of 6% to 18% among the eligible patients [3,12]. The main barriers are the lack of awareness, high prices, and the lack of multidisciplinary FP centers. These are particularly acute in low- and middle-income countries where resources and expertise are limited.

Increased access can be achieved by incorporating insurance of FP into health policies, creating regional oncofertility networks, and making fertility counseling a routine procedure in cancer treatment. Moreover, the oncology teams are to be trained in FP counselling, and patients are to be referred to reproductive specialists before the gonadotoxic therapy is started.

### 4.8. Need for Standardized Protocols and Follow-Up

One of the key issues that were found in the present research is the absence of standardized FP protocols. There is a great deal of difference in the duration of hormonal therapy, time of cryopreservation and definition of outcome. The treatment schedules were inconsistent, and the definitions of complete response and recurrence were not consistent [29,32]. Unified nomenclature and reporting systems, e.g., defining LBR per initiated FP cycle, are thus needed.

Moreover, there was inconsistency in long-term monitoring in studies. Although surgical and cryopreservation studies had more than six years of follow-up, hormone-therapy and transposition studies used shorter follow-up. The creation of a global registry that connects oncologic and reproductive data would increase the consistency and reliability of evidence.

### 4.9. Comparative Analysis

This investigation complements earlier systematic reviews by combining empirical data on five overall themes, oncologic safety, technological improvements, psychosocial benefits, accessibility barriers, and the necessity of standardization and therefore provides a comprehensive evaluation of the fertility preservation (FP) that goes beyond the independent outcomes effect. The use of several quality-assessment tools also contributes to the strength of the methodology of the synthesis. Although minor differences are present, including the relatively lower live birth rate (LBR) in (Fraison et al. 2023) [26], which is linked to demographic differences and the differences in the rate of FP utilization, the general findings are congruent across research (Fraison et al. 2023) [26]. One scientific deficiency that has been frequently identified among all of the reviews is the lack of longitudinal obstetric and offspring-level data, alongside discrepant reports of outcomes. The current research adds to the field because it supports the use of standardization models and the importance of integrating patient-focused, effective care paradigms to bridge the gap between the results of quantitative research and real-life scenarios of clinical use.

### 4.10. Clinical Implications

The current study proves that FP interventions are safe and effective in women with pelvic oncologic surgery. Quantitative evidence shows strong oncologic safety and positive reproductive results, and thematic results underline the significance of multidisciplinary collaboration, accessibility, and psychosocial support. Nevertheless, the issues of fluctuating access, non-standardization, and inadequate long-term data remain. Future research ought to focus on multicenter prospective studies that have a single endpoint and long-term follow-up to assess obstetric and offspring outcomes. Enhancement of global cooperation and policy advocacy may turn FP into a universal and fair part of cancer treatment in women of reproductive age.

### 4.11. Clinical Utility and Decision Pathway for Fertility Preservation

To improve clinical applicability, fertility preservation in women with gynecologic malignancies should follow a structured decision-making process integrating tumor type, disease stage, and planned oncologic treatment. Based on the current evidence, we propose a practical clinical decision pathway to support individualized counseling and management.

In early-stage cervical cancer, fertility-sparing surgery may be considered in carefully selected patients, whereas cryopreservation strategies and ovarian transposition should be discussed when gonadotoxic therapy is planned. In early-stage ovarian cancer, fertility-sparing surgery may be appropriate for selected low-risk cases, while cryopreservation options are recommended when adjuvant chemotherapy is required. Patients with advanced-stage disease or high-risk histology should be primarily counseled toward cryopreservation approaches. In early-stage endometrial cancer, conservative hormonal management may be considered in eligible patients, with fertility preservation strategies discussed prior to treatment initiation.

**Figure 5:**
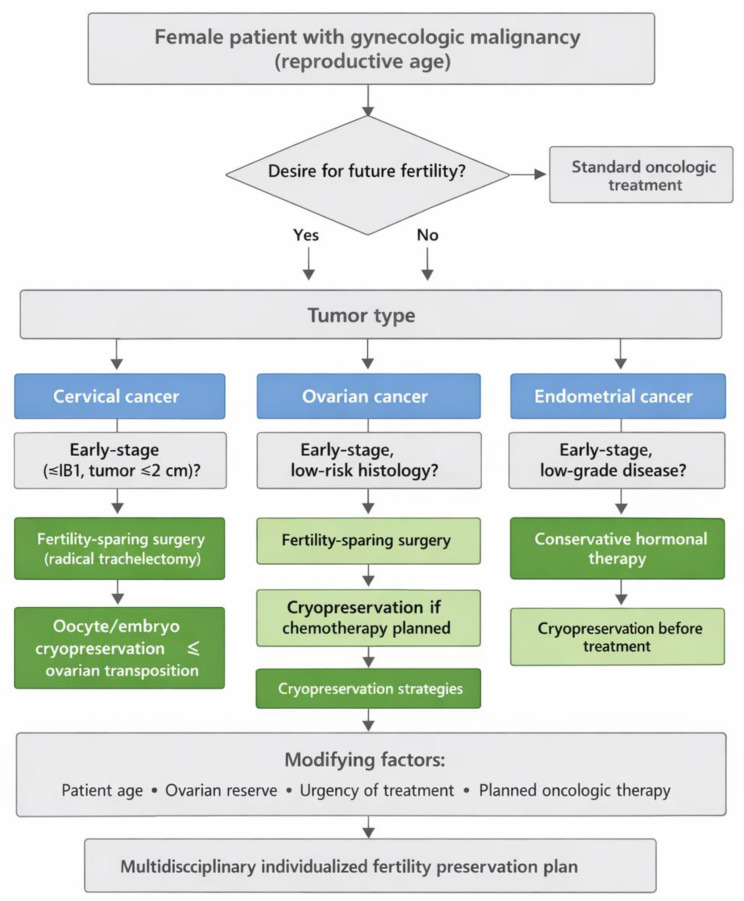
Clinical decision pathway for fertility preservation in women with gynecologic malignancies.

Across all tumor types, decision-making is influenced by patient age, ovarian reserve, and urgency of cancer treatment. A summary of this evidence-based approach is provided in Figure 5, which presents a visual clinical flowchart outlining fertility preservation options according to clinical scenario and treatment plan.

### 4.12. Emerging Technologies and Future Directions

Recent advances in reproductive medicine and oncofertility have significantly broadened the landscape of fertility preservation beyond traditional surgical and cryopreservation approaches. Ovarian tissue cryopreservation (OTC) has transitioned from an experimental technique to an increasingly utilized option, particularly for patients who cannot delay gonadotoxic cancer therapy or those in prepubertal age groups. Recent narrative reviews emphasize that OTC not only preserves fertility potential but also holds promise for restoring endocrine function, with emerging data on optimal cryopreservation and transplantation protocols that incorporate both slow-freezing and vitrification methods [46,47].

While OTC has shown encouraging clinical results, challenges persist with respect to long-term graft function, ischemia-related follicular loss, and the standardization of transplantation protocols. Continued refinement of surgical and laboratory techniques—including efforts to enhance vascularization and reduce apoptotic follicle loss—remains a priority in ongoing research [46].

Uterus transplantation (UTx) represents another frontier in fertility restoration for women affected by absolute uterine factor infertility. UTx has now evolved into a viable reproductive option, with a growing number of successful live births reported in recent years. Despite these advances, UTx continues to present complex clinical and ethical challenges, including the inherent morbidity associated with donor and recipient surgery, the need for prolonged immunosuppression, and considerations related to equitable access and resource allocation. These realities necessitate multidisciplinary care models that integrate transplant surgeons, reproductive endocrinologists, ethicists, and psychosocial support specialists to optimize patient outcomes and ensure responsible clinical implementation [48].

From a health policy perspective, the integration of emerging fertility preservation technologies into routine oncologic care requires not only robust clinical evidence but also standardized guidelines, cost-effectiveness analyses, and equitable care frameworks. Addressing disparities in access and ensuring informed, patient-centered decision-making will be critical to translating scientific advances into broad clinical benefit. This systematic review was conducted according to the PRISMA 2020 guidelines. Detailed data are available in Supplementary Materials [49].

## 5. Conclusions

Fertility preservation represents a critical component in safeguarding the reproductive potential of women undergoing pelvic oncologic surgery, while supporting reproductive autonomy without compromising oncologic outcomes. The present paper clarifies that FP methods do not only protect ovarian activity and reproductive potential but that they also present significant psychosocial advantages as they scrub not only the emotional but also the mental state of the patients. Surgery and cryopreservation modalities have proven to preserve oncologic safety so far without increasing the likelihood of recurrence hence enhancing reproductive autonomy without the need to sacrifice survival.

Fertility preservation following pelvic oncologic surgery requires careful consideration of factors influencing postoperative ovarian function in order to optimize reproductive outcomes.

Integrating predictive biomarkers into clinical decision-making may support more individualized fertility preservation planning and improved survivorship outcomes.

Overall, these findings indicate that fertility preservation strategies empower women to maintain reproductive potential while emphasizing the importance of multidisciplinary approaches within contemporary oncologic care.

## 6. Strengths and Limitations

This review integrates FP interventions of women experiencing surgery in cancer of the pelvis, and the strengths of this work include its multidisciplinary background of including surgical, reproductive, and oncologic viewpoints, which provide clinically relevant information that can be used in practical oncologic practice. The given analysis covers various research designs, such as systematic reviews, meta-analyses, and population-based cohorts, which ensure the finding of high robustness and generalizability. The review provides practical understanding on how to make surgical decisions and counsel patients by aligning the FP modalities with the contributory reproductive risk factors, namely, organ resections, radiation-induced gonadotoxicity, and chemotherapy-related ovarian failure. It also offers a logical conceptual framework that strikes a balance between oncologic safety, technological advancements and patient outcome. Nevertheless, though inherent to studies, the management of bias in this review is achieved by performing extensive appraisal processes, and an open-software synthesis model, concluding with the solutions based on the best of evidence and not determined by single methodological limitations.

This research received no specific grant from any funding agency in the public, commercial, or not-for-profit sectors.

## Figures and Tables

**Table 4:** AGREE II assessment of included clinical practice guidelines.

Study (Authors, Year)	Scope and Purpose	Stakeholder Involvement	Rigor of Development	Clarity of Presentation	Applicability	Editorial Independence	Mean Score (1–7)	Quality Grade	Key Comments
(Rodolakis et al., [29]	7	6	7	6	5	6	6.2	High	Strong ESGO/ESHRE/ESGE collaboration; transparent evidence grading.
(Su et al., [12])	7	7	7	7	6	6	6.7	High	ASCO methodological rigor and independence clearly demonstrated.

**Table 5:** Summary of included studies according to the PICO framework (population, intervention, comparison, outcomes).

Study (Authors, Year)	Population (P)	Intervention/Exposure (I)	Comparison (C)	Outcomes (O)
(Fraison et al., [26])	Women with pelvic malignancies undergoing fertility preservation	Oocyte, embryo, and ovarian tissue cryopreservation	No FP / conventional treatment	LBR 32–41%; no oncologic risk
(Nezhat et al., [23])	Early-stage cervical cancer (IA1–IB1)	Radical vaginal trachelectomy	Radical hysterectomy	Comparable OS/DFS; preserved fertility
(Li et al., [27])	Early-stage epithelial ovarian cancer	Fertility-sparing surgery (FSS)	Standard oophorectomy	LBR > 80%; OS 95–100%; recurrence 3–12%
(Liu et al., [28])	Cervical cancer stage IA–IB1	Vaginal radical trachelectomy	Radical hysterectomy	DFS 94–99%; similar recurrence
(Rodolakis et al., [29]	Early-stage endometrial carcinoma (IA, G1)	High-dose progestin therapy	Definitive hysterectomy	CR 66–91%; recurrence ~27%; pregnancy ~39%
(Genovese et al., [1])	Women receiving pelvic RT	Ovarian transposition	No ovarian preservation	61.7–100% ovarian function preserved
(Moawad et al., [30])	Gynecologic malignancy requiring pelvic RT	Laparoscopic ovarian transposition	None (historical control)	75% endocrine function retained
(Guo et al., [31])	Cervical cancer patients (IA1–IB1)	Radical trachelectomy	Radical hysterectomy	5 yr DFS 97–99%; preserved fertility
(Park et al., [32])	Early-stage endometrial carcinoma	Oral progestin ± LNG-IUS	No fertility preservation	CR 79.7%; pregnancy 39.1%; recurrence 27.5%
(Xu et al., [33])	Female cancer survivors	Embryo and oocyte cryopreservation	No FP	Pooled LBR 32–41%; safe pre-treatment
(Cobo et al., [34])	Oncology patients preserving fertility	Oocyte vitrification	Fresh IVF cycles	LBR 35%; comparable safety
(Morice et al., [24])	Early-stage cervical cancer	Radical trachelectomy (FSS)	Standard hysterectomy	5 yr OS 97–100%; comparable DFS
(Su et al., [12])	Women with cancer eligible for FP	Multimodal FP (cryo + surgery)	No FP/standard care	High safety; improved reproductive outcomes
(Gică et al., [3])	Pelvic malignancies; FP-eligible	Combined FP (OTC, oocyte, surgery)	Conventional care	Safe integration; 8% cumulative LBR; low recurrence

**Table 6:** Thematic coding of findings from included studies.

Code ID	Initial Code (Descriptive Phrase)	Supporting Evidence (Study ID and Quote)	Frequency Across Studies	Interpretation (Analytic Note)
C1	Comparable survival after fertility-sparing surgery	2, 3, 4, 8, 12, 13: “No significant difference in 5-year OS or DFS”	6	Fertility-sparing surgery maintains oncologic safety comparable to standard radical procedures.
C2	Cryopreservation preserves ovarian function in majority	1, 6, 10, 11: “Ovarian function preserved 61.7–94% after transposition/cryopreservation”	4	Cryopreservation strategies effectively maintain both endocrine and reproductive function.
C3	Lack of standardization in hormonal therapy duration	5, 9: “Varied progestin dosing/duration and response rates”	2	Hormonal fertility preservation protocols require unified duration and dosage standards.
C4	Psychosocial benefits of fertility preservation	14: “Fertility preservation improves quality of life and emotional well-being”	1	Fertility preservation enhances psychological resilience and post-treatment quality of life.
C5	Multidisciplinary counseling improves uptake	5, 13, 14: “Early referral and multidisciplinary care increase FP utilization”	3	Collaborative care models support informed decision-making and improve fertility preservation uptake.
C6	Underutilization in low-resource settings	14: “Low utilization (6–18%) partly due to cost, provider knowledge, and resource gaps”	1	Fertility preservation remains underutilized in low- and middle-income countries due to systemic barriers.
C7	No evidence of increased recurrence risk post-FP	1, 2, 3, 4, 10, 13: “Recurrence rates low, comparable to controls”	6	Fertility preservation interventions are oncologically safe and do not increase recurrence risk.
C8	Tumor size and histology as prognostic factors	2, 12: “Higher recurrence in tumors >2 cm; histology influences FP safety”	2	Oncologic outcomes depend on tumor stage, histological subtype, and margin status, guiding patient selection.

**Table 7:** Final themes and interpretive summaries.

Theme	Description	Evidence Strength	Key Supporting Studies	Implications/Novel Contributions
1. Oncologic Safety of Fertility Preservation Procedures	Fertility preserving procedures in cervical, ovarian and endometrial cancers have shown similar recurrence and survival results to conventional radical procedures in well-selected patients.	High	Fraison et al. (2023) [26]; Nezhat et al. [23]; Li [21]; Liu [28]; Guo [31]; Morice (2022) [24]; Su (2025) [12]	Confirms that FP does not increase oncologic risk, supporting its inclusion in treatment planning for early-stage cancers. Reinforces oncologic–reproductive coexistence as a viable care paradigm.
2. Evolving Techniques and Multimodality Integration	Advancements such as ovarian tissue cryopreservation, uterine transposition, and minimally invasive trachelectomy provide safe, adaptable fertility options. Integration with ART and endocrine preservation has enhanced reproductive outcomes without compromising safety.	High	Genovese et al. [1]; Moawad et al. [30]; Cobo (2016) [34]; Xu [33]); Gica (2025) [3]	Demonstrates growing feasibility of multimodal FP; highlights innovation in surgical and cryobiological methods as key drivers of reproductive success.
3. Long-Term Reproductive and Psychosocial Outcomes	Fertility preservation interventions restore reproductive potential and improve psychological well-being, autonomy, and long-term quality of life for cancer survivors.	Moderate	Fraison (2023 [26]); Rodolakis [29]; Su (2025) [12]; Gica (2025) [3]	Determines FP dual role of physiological recovery and emotional strength; facilitates psychosocial aspects of FP counseling and outcome measures.
4. Barriers to Access and Utilization	FP remains underutilized globally due to costs and resources limitations, low referral rates, and inadequate awareness among providers and patients—especially in low- and middle- income settings.	Moderate	Gica (2025) [3]; Su (2025) [12]; Rodolakis [29]	Recognizes structural injustices and the need of health policy measures and resources allocation to increase FP access.
5. Need for Standardized Protocols and Follow-up	There is a wide level of heterogeneity in hormonal therapy time, follow-up, and reproductive and oncologic endpoint reporting in the FP literature. This restricts meta-analytic comparability and evidence synthesis.	Moderate	Rodolakis [29]; Park [32]; Morice (2022) [24]; Gica (2025 [3])	Reports urgent necessity of standardized FP protocols, single outcome definitions and international registries-based follow-up systems to enhance data comparability and evidence quality.

## Data Availability

As this is a systematic review and meta-analysis, no new primary data were generated during this study. All data analyzed in this review were extracted from previously published studies, which are cited in the References section. The data supporting the findings of this systematic review are available in the published articles referenced in the manuscript and in the Supplementary Materials (PRISMA 2020 Checklist).

## Supplementary Materials

The following supporting information can be downloaded at: https://www.mdpi.com/article/10.3390/cancers18071142/s1, PRISMA 2020 Checklist [49].

## Abbreviations

The following abbreviations are used in this manuscript:

FP	Fertility Preservation
FSS	Fertility-Sparing Surgery
OTC	Ovarian Tissue Cryopreservation
RT	Radiotherapy
CPR	Clinical Pregnancy Rate
LBR	Live Birth Rate
OS	Overall Survival
DFS	Disease-Free Survival
PRISMA	Preferred Reporting Items for Systematic Reviews and Meta-Analyses
PICO	Population, Intervention, Comparator, Outcome
NOS	Newcastle–Ottawa Scale
AMSTAR 2	A Measurement Tool to Assess Systematic Reviews 2
ASCO	American Society of Clinical Oncology
ESGO	European Society of Gynaecological Oncology
ESHRE	European Society of Human Reproduction and Embryology
ESGE	European Society for Gynaecological Endoscopy
IVF	In Vitro Fertilization
OT	Ovarian Transposition
CR	Complete Response
LMICs	Low- and Middle-Income Countries
QoL	Quality of Life
