# Supplementary material for: Fertility Preservation Strategies in Women with Pelvic Gynecologic Malignancies Undergoing Multimodal Oncologic Treatment: A Systematic Review
Source: Cancers (Basel). 2026 Apr 2;18(7):1142. doi: 10.3390/cancers18071142 (PMC13072090; doi:10.3390/cancers18071142)
Supplement: Supplementary file 1 [file cancers-18-01142-s001.zip › cancers-4039578-supplementary.pdf]

PRISMA 2020 Checklist

Section & Topic	Item #	Checklist Item	Location in Manuscript
TITLE			
Title	1	Identify the report as a systematic review.	Title page (“A Systematic Review”)
ABSTRACT			
Abstract	2	Follow PRISMA 2020 for Abstracts checklist.	Abstract section (structured)
INTRODUCTION			
Rationale	3	Describe rationale in context of existing knowledge.	Introduction, paragraphs 1–4
Objectives	4	State objectives/questions addressed.	End of Introduction, final paragraph
METHODS			
Eligibility criteria	5	Inclusion/exclusion criteria; how studies grouped.	Methods → “Eligibility Criteria”
Information sources	6	All databases, registers, websites; last search date.	Methods → “Information Sources”
Search strategy	7	Full search strategies for all databases.	Methods → “Search Strategy”
Selection process	8	Reviewers involved, independence, tools.	Methods → “Study Selection”
Data collection process	9	Reviewers, independence, procedures for confirming data.	Methods → “Data Extraction”
Data items	10a	Outcomes sought and how decided.	Methods → “Data Extraction” & Table 5

PRISMA

PRISMA 2020 Checklist

Section & Topic	Item #	Checklist Item	Location in Manuscript
	10b	Other variables (participant/intervention characteristics, assumptions).	Methods → "Data Extraction"; Study Characteristics section
Study risk of bias assessment	11	Methods, tools used, number of reviewers, independence.	Methods → "Quality Appraisal"
Effect measures	12	Effect measures used.	Methods → "Application of PICO framework"
Synthesis methods	13a	How studies were selected for each synthesis.	Methods → "Eligibility Criteria" + "Study Selection"
	13b	Data preparation, handling missing data.	Methods → "Data Extraction"
	13c	Tabulation/visual display methods.	Methods → Figures 2–4; Tables 1–7
	13d	Synthesis rationale; models if meta-analysis.	Not applicable (narrative/thematic synthesis only)
	13e	Explorations of heterogeneity.	Narrative exploration in Discussion → comparative analysis
	13f	Sensitivity analyses.	Not applicable (qualitative synthesis only)
Reporting bias assessment	14	Methods to assess reporting bias.	Methods → "Quality

PRISMA

PRISMA 2020 Checklist

Section & Topic	Item #	Checklist Item	Location in Manuscript
			Appraisal"; addressed indirectly via AMSTAR 2/NOS/AGREE II
Certainty assessment	15	Methods to assess certainty.	Methods → "Quality Appraisal"; PICO application
RESULTS			
Study selection	16a	Describe results of search and selection process.	Results → "Study Selection"; PRISMA flow diagram (Figure 1)
	16b	Cite studies excluded and reasons.	Results → "Study Selection" (exclusion reasons listed)
Study characteristics	17	Present characteristics of each study.	Results → "Study Characteristics"; Table 1
Risk of bias in studies	18	Present bias assessments.	Results → "Quality Assessment"; Tables 2–4
Results of individual studies	19	Summary statistics and effect estimates per study.	Results → Table 1; PICO table; narrative summaries
Results of	20a	Summaries of characteristics/risk of bias.	Results → "Quality Assessment";

Section & Topic	Item #	Checklist Item	Location in Manuscript
syntheses			Thematic Synthesis
	20b	Statistical syntheses results.	Not applicable (no meta-analysis)
	20c	Heterogeneity investigations.	Discussion → Comparative Analysis
	20d	Sensitivity analyses.	Not applicable
Reporting biases	21	Bias due to missing results.	Results → "Quality Assessment"; addressed via AMSTAR 2/NOS
Certainty of evidence	22	Present certainty/confidence per outcome.	Results → "Quality Assessment"; PICO framework
DISCUSSION			
Interpretation	23a	Interpretation in context of evidence.	Discussion → first three paragraphs
Limitations of evidence	23b	Limitations of included evidence.	Discussion → "Barriers to Access…" + "Need for Standardized…"
Limitations of review processes	23c	Limitations of the review methods.	Discussion → final paragraphs before Comparative Analysis
Implications	23d	Practice, policy, future research.	Discussion → "Clinical Implications"
OTHER INFORMATION			

PRISMA

PRISMA 2020 Checklist

Section & Topic	Item #	Checklist Item	Location in Manuscript
Registration and protocol<br>24c	24a	Registration information.	Not registered (add: "This review was not prospectively registered.")
	24b	Protocol access.	No protocol prepared. Add to Methods.
	Amendments to protocol.	Not applicable.	
Support	25	Funding sources and role.	Declarations → "Funding"
Competing interests	26	Competing interests.	Declarations → "Competing interests"
Availability of data, code, materials	27	What is publicly available & where.	Declarations → Data availability

From: Page MJ, McKenzie JE, Bossuyt PM, Boutron I, Hoffmann TC, Mulrow CD, et al. The PRISMA 2020 statement: an updated guideline for reporting systematic reviews. BMJ 2021;372:n71. doi: 10.1136/bmj.n71. This work is licensed under CC BY 4.0. To view a copy of this license, visit https://creativecommons.org/licenses/by/4.0/
